# The isothiocyanate sulforaphane inhibits mTOR in an NRF2-independent manner

**DOI:** 10.1016/j.phymed.2019.153062

**Published:** 2021-06

**Authors:** Ying Zhang, Amy Gilmour, Young-Hoon Ahn, Laureano de la Vega, Albena T. Dinkova-Kostova

**Affiliations:** a Jacqui Wood Cancer Centre, Division of Cellular Medicine, School of Medicine, University of Dundee, Dundee, Scotland DD1 9SY, United Kingdom; b Department of Chemistry, Wayne State University, Detroit, MI, United States; c Department of Pharmacology and Molecular Sciences and Department of Medicine, Johns Hopkins University School of Medicine, Baltimore, MD 21205, United States

**Keywords:** HDAC6, mTOR, NRF2, PI3K-AKT, Sulforaphane, AMPK, 5′-AMP-activated protein kinase, BITC, benzyl isothiocyanate, CBHA, m-carboxycinnamic acid bis-hydroxamide, HDAC, histone deacetylase, KEAP1, Kelch-like ECH-associated protein 1, NRF2, nuclear factor erythroid 2 p45-related factor 2, mTOR, mechanistic target of rapamycin, NRF2, nuclear factor erythroid 2 p45-related factor 2, PI3K-AKT, phosphatidylinositol 3-kinase-AKT/protein kinase B, SFN, sulforaphane

## Abstract

**Background:**

The isothiocyanate sulforaphane (SFN) has multiple protein targets in mammalian cells, affecting processes of fundamental importance for the maintenance of cellular homeostasis, among which are those regulated by the stress response transcription factor nuclear factor erythroid 2 p45-related factor 2 (NRF2) and the serine/threonine protein kinase mechanistic target of rapamycin (mTOR). Whereas the way by which SFN activates NRF2 is well established, the molecular mechanism(s) of how SFN inhibits mTOR is not understood.

**Hypothesis/Purpose:**

The aim of this study was to investigate the mechanism(s) by which SFN inhibits mTOR

**Study design and methods:**

We used the human osteosarcoma cell line U2OS and its CRISPR/Cas9-generated NRF2-knockout counterpart to test the requirement for NRF2 and the involvement of mTOR regulators in the SFN-mediated inhibition of mTOR.

**Results:**

SFN inhibits mTOR in a concentration- and time-dependent manner, and this inhibition occurs in the presence or in the absence of NRF2. The phosphatidylinositol 3-kinase (PI3K)-AKT/protein kinase B (PKB) is a positive regulator of mTOR, and treatment with SFN caused an increase in the phosphorylation of AKT at T308 and S473, two phosphorylation sites associated with AKT activation. Interestingly however, the levels of pS552 β-catenin, an AKT phosphorylation site, were decreased, suggesting that the catalytic activity of AKT was inhibited. In addition, SFN inhibited the activity of the cytoplasmic histone deacetylase 6 (HDAC6), the inhibition of which has been reported to promote the acetylation and decreases the kinase activity of AKT.

**Conclusion:**

SFN inhibits HDAC6 and decreases the catalytic activity of AKT, and this partially explains the mechanism by which SFN inhibits mTOR.

## Graphical abstract

**Figure:**
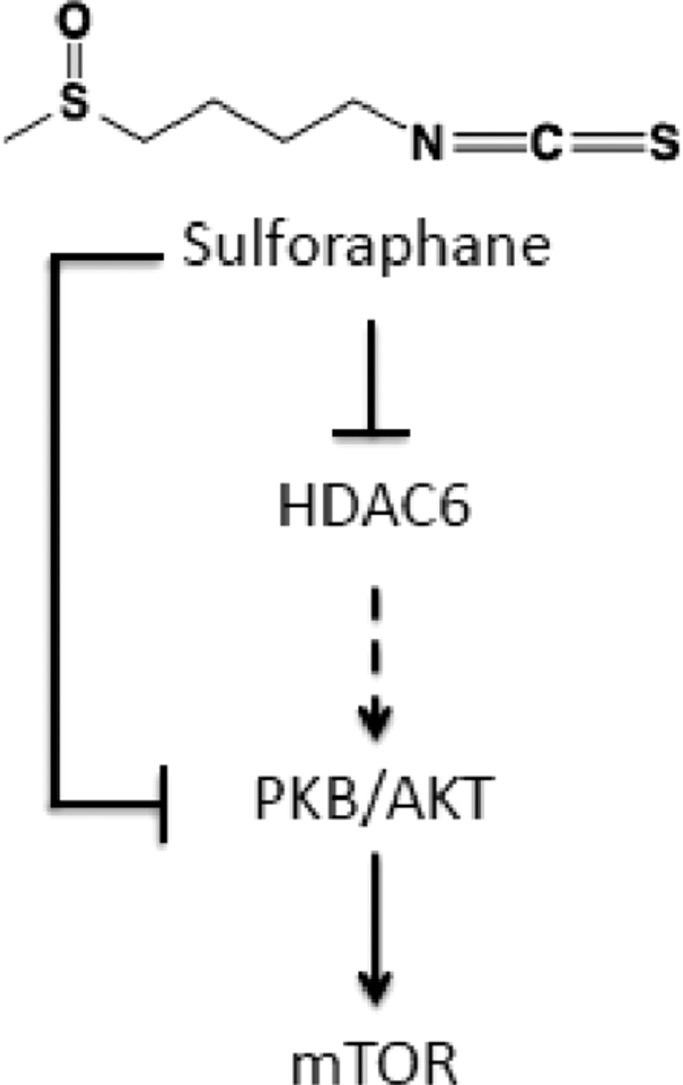

## Introduction

**Figure 1:**
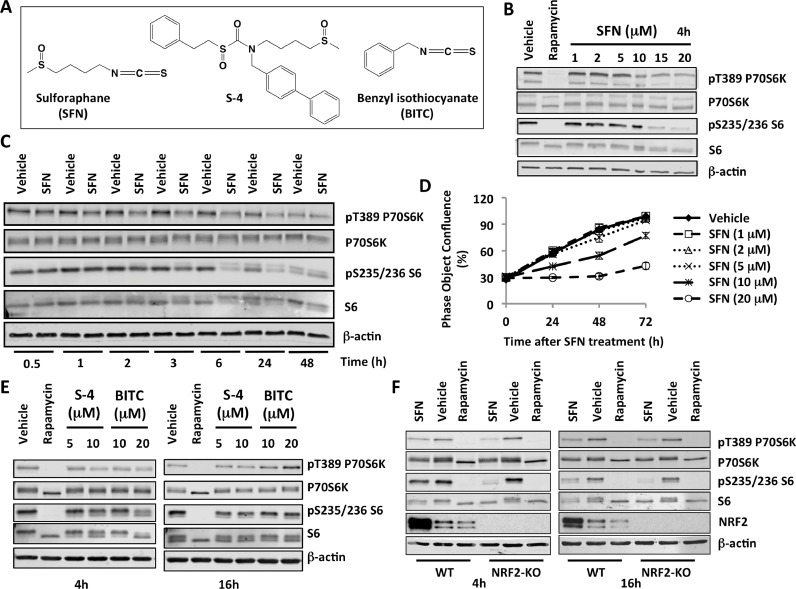
**Sulforaphane (SFN) inhibits mTOR in an NRF2-independent manner. (A)** Chemical structures of the NRF2 activators used in this study. **(B,C,E,F)** Immunoblotting analysis of phosphorylated p70S6K (T389), p70S6K, phosphorylated S6 (S235/236), and S6 in lysates from U2OS cells, which had been treated with vehicle (0.1% DMSO), rapamycin (20 nM), SFN (20 μM, unless otherwise indicated) **(B,C,F)**, S-4 **(E)**, or BITC **(E)** for the specified periods of time. The levels of β-actin served as a loading control. **(D)** Cell proliferation in the presence of increasing concentrations of SFN.

The isothiocyanate sulforaphane (SFN) (Fig. 1A) is a hydrolytic product of the glucosinolate glucoraphanin, a phytochemical present in cruciferous vegetables, which are widely known for their chemopreventive properties. In contrast to its chemically inert precursor, SFN is highly reactive due to the presence of an electrophilic carbon within the isothiocyanate (—N=C=S) group. Multiple protein targets for SFN have been identified in mammalian cells, with Kelch-like ECH-associated protein 1/nuclear factor erythroid 2 p45-related factor 2 (KEAP1/NRF2) pathway being the most-studied and best-understood SFN target (Dinkova-Kostova et al., 2017). SFN chemically modifies C151 within the BTB (*B*road-Complex, *T*ramtrack, and *B*ric à brac) dimerization domain of KEAP1, the main negative regulator of transcription factor NRF2, leading to NRF2 activation and enhanced expression of a large battery of cytoprotective proteins (Zhang and Hannink, 2003).

The mechanistic target of rapamycin (mTOR) is a serine/threonine protein kinase. mTOR is the catalytic subunit of two distinct protein complexes, known as mTOR Complex 1 (mTORC1) and 2 (mTORC2), which have fundamental roles in the maintenance of cellular homeostasis by coordinating cell growth and metabolism with environmental inputs (Saxton and Sabatini, 2017). Dysregulation of mTOR is associated with numerous human diseases, such as type 2 diabetes, cancer, and neurodegeneration, and the development of mTOR inhibitors is actively being pursued, particularly in oncology (Saxton and Sabatini, 2017). A number of studies have shown that SFN affects mTOR signalling. Thus, in breast and prostate cancer cell lines, SFN suppresses mTOR and induces autophagy (Wiczk et al., 2012). A recent study in human vascular smooth muscle cells suggests that the SFN-mediated inhibition of mTOR is independent of NRF2 activation (Shawky and Segar, 2017). However, the molecular mechanism of how SFN inhibits mTOR has not been addressed.

SFN has multiple protein targets. Thus, SFN inhibits histone deacetylase (HDAC) activity (Myzak et al., 2006), and one study found that the levels of phosphorylated mTOR substrates are lower in the absence of HDAC6, a cytoplasmic protein deacetylase (Moreno-Gonzalo et al., 2017). SFN was also reported to activate 5′-AMP-activated protein kinase (AMPK) (Yang et al., 2016), a negative regulator of mTOR, and to affect (either inhibit or stimulate) phosphatidylinositol 3-kinase (PI3K)-AKT/protein kinase B (PKB) (Shen et al., 2007, Wu et al., 2019), a positive regulator of mTOR. Together, these studies suggest multiple possible mechanisms by which SFN inhibits mTOR.

To address these possibilities, as well as the potential involvement of NRF2 in the SFN-mediated inhibition of mTOR, in this study we used the human osteosarcoma cell line U2OS and its CRISPR/Cas9-generated NRF2-knockout counterpart. We chose human osteosarcoma cells as a model system, because SFN has been previously shown to: (i) induce apoptosis and enhance the radiosensitivity of LM8 murine osteosarcoma cells, and inhibit tumor LM8 xenograft growth (Matsui et al., 2007, Sawai et al., 2013); (ii) promote apoptosis in the human osteosarcoma cell lines U2OS, Saos2 and MG63 (Ferreira de Oliveira et al., 2014, Kim et al., 2011, Matsui et al., 2007, 2006); (iii) inhibit cell migration and angiogenesis in U2OS xenografts (Jeong et al., 2017).

## Materials and methods

### Materials

*R,S*-Sulforaphane (SFN) was from LKT Laboratories. S-4 was synthesized as described (Zhang et al., 2014). Benzyl isothiocyanate (BITC) and rapamycin were from Sigma-Aldrich. Tubastatin A was from BioVision. MS-275 and m-carboxycinnamic acid bis-hydroxamide (CBHA) were from Santa Cruz Biotechnology. The compounds were dissolved in DMSO (vehicle) and diluted in the cell culture medium such that the final DMSO concentration was maintained at 0.1%. The concentrations of each compound are stated in the figure legends.

### Cell culture

Human osteosarcoma (U2OS) cells were cultured in Dulbecco's Modified Eagle Medium (DMEM) that contains L-glutamine, sodium pyruvate, and D-glucose (4.5 g/L), supplemented with 10% (v/v) fetal bovine serum (FBS). NRF2-knockout (NRF2-KO) U2OS cells with homozygous knockout of the *NFE2L2* gene, which encodes NRF2, were produced by transfecting U2OS cells with pLentiCRISPRv2 plasmid (a gift from Dr Feng Zhang, Addgene plasmid #52961, which encodes Cas9 and a puromycin cassette) containing a guide RNA directed against exon 2 of the *NFE2L2* locus (5′-TGGAGGCAAGATATAGATCT-3′). After two days of puromycin selection, cells were clonally selected by serial dilution, and positive clones were identified as described (Torrente et al., 2017). Control cells, referred as U2OS wild-type (WT), were the pooled population of surviving cells transfected with an empty pLentiCRISPRv2 vector and treated with puromycin. Cells were maintained in a humidified atmosphere at 37 °C and 5% CO_2_. For immunoblotting experiments, cells were seeded into 6-well plates at a density of 3 × 10^5^ per well the day before treatments. For overexpression of HDAC6, 1 μg of empty vector or a plasmid encoding FLAG-HDAC6 [a gift from Dr XJ Yang (Zhang et al., 2007)] was transfected using Lipofectamine-2000 (Invitrogen).

### Cell proliferation

Cell proliferation was assessed using an IncuCyte Zoom live cell imaging microscope (Essen Bioscience, Welwyn Garden City, UK) with 10× objective and data management software. Cells were seeded at a density of 5 × 10^3^ cells per well in 96-well plates. On the next day, cells were treated with the indicated concentrations of SFN once, and the confluence of the cell culture was monitored by IncuCyte Zoom imaging over a period of 72 h. Data presented are the mean ± S.D. of 6 replicate wells taken from a representative experiment.

### Immunoblotting

Cells were lysed in 150 µl of non-reducing sample buffer (50 mM Tris–HCl pH 6.8, 2% (w/v) sodium dodecyl sulfate (SDS), 10% (v/v) glycerol, and 0.02% (w/v) Bromophenol blue). Whole-cell lysates were then collected in Eppendorf tubes, boiled at 100 °C for 5 min, and sonicated using Vibra-Cell ultrasonic processor (Sonic) for 20 s at 20% amplitude. The BCA assay (Thermo) was used to determine protein concentrations. 2-Mercaptoethanol (Sigma) was added to a final concentration of 6% (v/v). Proteins were resolved using 10% SDS-PAGE, and transferred to 0.45 µm nitrocellulose (NC) membranes.

Solutions of primary antibodies were prepared in 3% BSA (antibodies against phospho-proteins) or 5% milk (all other) in PBST. The following antibodies were used: rabbit monoclonal anti-phospho-p70 S6K (T389), 1:1000, CST; rabbit monoclonal anti-p70 S6K, 1:1000, CST; rabbit monoclonal anti-phospho-S6(S235/6), 1:2000, CST; mouse monoclonal anti-S6, 1: 1000, CST; rabbit monoclonal anti-phospho-AKT (S473), 1: 1000, CST; rabbit monoclonal anti-phospho-AKT (T308), 1: 1000, CST; rabbit monoclonal anti-AKT(pan), 1: 1000, CST; rabbit monoclonal anti-Ac-α-tubulin (K40), 1:1000, CST; mouse monoclonal anti-histone H3, 1:1000, CST; rabbit polyclonal anti-Ac-histone H3 (K9), 1:1000, Active Motif; rabbit monoclonal anti-Ac-histone H3 (K27), 1:1000, CST; mouse monoclonal anti-α-tubulin, 1: 1000, CST; rabbit monoclonal anti-phospho-AMPKα(T172), 1: 1000, CST; rabbit monoclonal anti-AMPKα, 1: 1000, CST; rabbit monoclonal anti-NRF2, 1: 1000, CST; rabbit monoclonal anti-FLAG tag, 1:1000, CST; rabbit polyclonal anti-HDAC6, 1:200, Santa Cruz; rabbit polyclonal anti-phospho β-catenin (S552), 1:1000, CST; rabbit polyclonal anti-β-catenin, 1:1000, CST; mouse monoclonal anti-β-actin antibody, 1:20 000, Sigma. Blocked NC membranes were incubated with the primary antibody solutions in 50-ml tubes at 4 °C overnight, with continuous rotation. After this, the NC membranes were washed and incubated with the corresponding secondary antibodies (horseradish peroxidase (HRP)-conjugated goat anti-rabbit (GAR) antibody, 1:5000, Bio-Rad or IRDye fluorescent dyes conjugated GAM-680RD or GAM/GAR-800CW, 1:15000, LI-COR). All blots are representative of at least three independent experiments.

## Results and discussion

### Sulforaphane inhibits mTOR in an NRF2-independent manner

mTOR phosphorylates ribosomal p70S6 kinase 1 (p70S6K) at its hydrophobic motif site, T389; in turn, the phosphorylated p70S6K phosphorylates its substrate, the S6 ribosomal protein at S235/236. Exposure of U2OS cells to increasing concentrations of SFN caused a concentration-dependent reduction in the levels of phosphorylated p70S6K (T389) and S6 (S235/236) (Fig. 1B and Fig. S1), strongly suggesting inhibition of mTOR. This observation is consistent with the previously reported inhibition of mTOR by SFN in other experimental systems, such as primary murine hepatocytes (Yang et al., 2016), breast and prostate cancer cell lines (Wiczk et al., 2012), and human aortic vascular smooth muscle cells (Shawky and Segar, 2017).

The concentration of SFN that gave the most pronounced effect on mTOR inhibition was 20 μM. Notably, this concentration is physiologically relevant. Thus, in a rat pharmacokinetic study of a single 150 μmol oral SFN dose, the peak plasma levels of SFN and its metabolites reached 60 μM 1 h after dosing, with a second peak of 22 μM at 1.5 h (Cornblatt et al., 2007). We therefore used 20 μM SFN in all subsequent studies. A time-course experiment showed that the inhibition of the phosphorylation of 70S6K occurred as early as 2 h of exposure to SFN (Fig. 1C and Fig. S2). The inhibition of the phosphorylation of the substrate, S6, followed, and was apparent at 3 h post-treatment. In agreement with the inhibition of mTOR, cell proliferation was inhibited by SFN in a concentration-dependent manner (Fig. 1D). Notably, the decrease in the levels of pT389 p70S6K and pS235/236 S6 was still evident at the 24 h- and even the 48 h time-point (Fig. 1C). The persistence of this effect of SFN is in agreement with the rapid accumulation and large area under the curve of intracellular concentration of SFN (and its metabolites) over a period of 24 h (Zhang and Talalay, 1998).

Next, using identical experimental conditions, we tested the possibility that two other compounds related to SFN may inhibit mTOR. Compound S-4 (Fig. 1A) is an analogue of SFN, where the strongly-electrophilic isothiocyanate group is replaced with the moderately-electrophilic sulfoxythiocarbamate group (Zhang et al., 2014). Like the isothiocyanates, such analogues target cysteine residues in proteins, but unlike the isothiocyanate, which react with sulfhydryl groups reversibly giving rise to kinetically labile dithiocarbamates, the sulfoxythiocarbamates form stable thiocarbamate adducts (Ahn et al., 2010). We also tested the aromatic benzyl-isothiocyanate (BITC, Fig. 1A), because it has higher sulfhydryl reactivity than SFN (Zhang et al., 1995). Similar to SFN, exposure to both S-4 and BITC for 4 h led to a decrease in the levels of pT389 p70S6K and pS235/236 S6 (Fig. 1E, left), indicating inhibition of mTOR. Of note, only rapamycin inhibited the phosphorylation of P70S6K and S6 completely as demonstrated by the absence of the top (presumably phosphorylated) bands corresponding to the total P70S6K and S6 proteins. In contrast to SFN, the mTOR inhibitory effect of S-4 and BITC, which was observed at 4 h post-exposure, was completely lost 16 h later (Fig. 1E, right). This difference in duration of mTOR inhibition between SFN and BITC correlates with the difference in metabolism and excretion of the two isothiocyanates (Zhang and Talalay, 1998), and suggests that persistent presence of the compound in the cell is required for long-lasting efficacy.

Considering that the KEAP1/NRF2 pathway is a major target for the isothiocyanates, we next tested the requirement for NRF2 for the SFN-mediated inhibition of mTOR. To do this, NRF2-knockout U2OS cells were generated using CRISPR/Cas9 genome editing. The levels of pT389 p70S6K and pS235/236 S6 were lower in both NRF2-wild-type- and NRF2-knockout SFN-treated cells compared to the corresponding vehicle-treated cells following 4 h as well as 16 h exposure to the isothiocyanate (Fig. 1F). Curiously, treatment with the classical mTOR inhibitor rapamycin that was used as a positive control caused a decrease in the protein levels of NRF2, demonstrating the high sensitivity of NRF2 to inhibition of protein synthesis. As expected, treatment with SFN increased the levels of NRF2. This experiment showed that, although SFN robustly activates NRF2, the ability of SFN to inhibit mTOR is not dependent on NRF2, in close agreement with recent findings that the SFN-mediated inhibition of mTOR in human aortic vascular smooth muscle cells still occurred following acute NRF2 knockdown by siRNA (Shawky and Segar, 2017).

### Sulforaphane causes transient inhibition of HDAC6 and AKT

**Figure 2:**
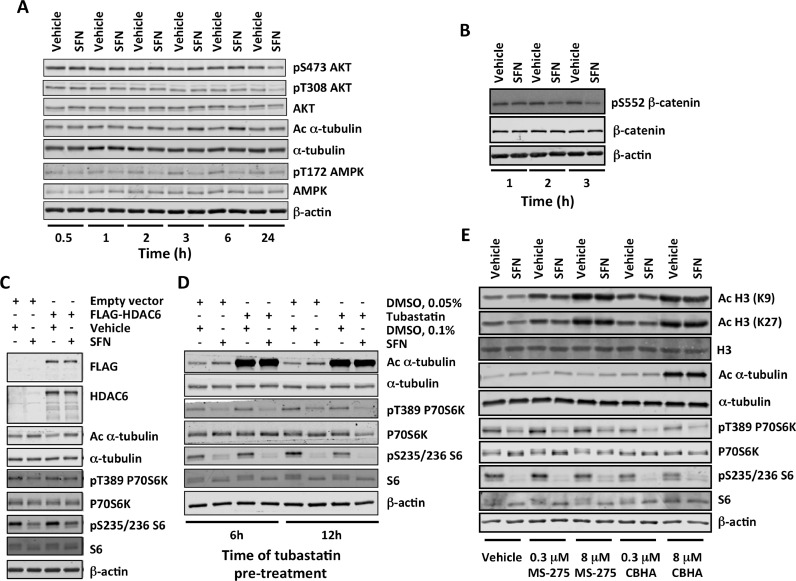
**Sulforaphane (SFN) causes transient inhibition of HDAC6 and AKT. (A,B)** U2OS cells were treated with vehicle (0.1% DMSO), SFN (20 μM) or rapamycin (20 nM) for the indicated periods of time. Immunoblotting analysis of whole-cell lysates was used to determine the levels of: phosphorylated AKT (S473 and T308), AKT, acetylated α-tubulin (K40), and α-tubulin **(A)**; phosphorylated AMPK (T172) and AMPK **(A)**; phosphorylated β-catenin (S552) and β-catenin **(B). (C)** Cells were transfected with an empty vector or a plasmid encoding FLAG-HDAC6. After 48 h, they were treated with SFN (20 μM) for a further 4 h. The levels of HDAC6, acetylated α-tubulin (K40), α-tubulin, phosphorylated p70S6K (T389), p70S6K, phosphorylated S6 (S235/236), and S6 were determined in whole-cell lysates by immunoblotting. **(D)** Following pre-treatment with tubastatin (5 μM) for the indicated periods of time, cells were treated with SFN (20 μM) for 4 h in the presence of tubastatin. The levels of acetylated α-tubulin (K40), α-tubulin, phosphorylated p70S6K (T389), p70S6K, phosphorylated S6 (S235/236), and S6 were determined in whole-cell lysates by immunoblotting. **(E)** Cells were pre-treated with the indicated concentrations of MS-275 or CBHA for 12 h, and subsequently treated with SFN (20 μM) for 4 h in the presence of the HDAC inhibitors. The levels of acetylated histone H3 (K9 and K27), histone H3, acetylated α-tubulin (K40), α-tubulin, phosphorylated p70S6K (T389), p70S6K, phosphorylated S6 (S235/236), and S6 were determined in whole-cell lysates by immunoblotting. In all cases, the levels of β-actin served as a loading control.

To address the mechanism by which SFN inhibits mTOR, we first examined the levels of AMPK phosphorylated at T172, a critical activating phosphorylation site located in the catalytic subunit of the enzyme (Hawley et al., 1996). The levels of pT172 AMPK were decreased by the SFN treatment (Fig. 2A). Since AMPK is a negative regulator of mTOR, this result ruled out the possibility that the mTOR inhibition by SFN could be due to AMPK activation.

We then turned our attention to AKT, a positive regulator of mTOR. This kinase phosphorylates mTOR at S2448 (Nave et al., 1999) as well as TSC2 within the tuberous sclerosis complex (TSC), which leads to TSC inhibition thereby relieving the TSC-mediated inhibition of Rheb, a potent direct activator of mTORC1 (Dibble and Manning, 2013). It has been shown that in human neural progenitor cells AKT interacts with HDAC6 (Iaconelli et al., 2017). Moreover, it has been demonstrated that a selective HDAC6 inhibitor (ACY-1215) promotes the acetylation and decreases the kinase activity of AKT, in spite of causing an increase in the levels of pT308 AKT and pS473 AKT (Iaconelli et al., 2017). Thus, it was possible that SFN, through its ability to inhibit HDAC6 (Myzak et al., 2006), causes a decrease in AKT catalytic activity. The levels of pT308 AKT and pS473 AKT decreased, but only 24 h after SFN treatment, and even slightly increased at the earlier time-points (Fig. 2A). Interestingly, rapamycin showed similar increase at all-time points (Fig. S3). Although surprising, this observation is consistent with published work showing that suppression of negative feedback loops mediated by mTORC1 causes compensatory activation of upstream regulators, including AKT (Rozengurt et al., 2014). Importantly however, the levels of pS552 β-catenin, an AKT phosphorylation site (Fang et al., 2007), were decreased by the SFN treatment (Fig. 2B), indicating that the catalytic activity of AKT was inhibited.

Treatment with SFN (Fig. 2A), but not rapamycin (Fig. S2), caused an increase in the acetylation of α-tubulin at K40, a classical HDAC6 substrate, confirming that HDAC6 was inhibited. The tubulin hyperacetylation was evident after 2, 3, 4, and 6 hours of SFN treatment (Fig. 2A,C,D,E). Notably, during this time period, the mRNA levels for HDAC6 were not affected by SFN (Fig. S4), indicating that the SFN treatment inhibits the activity of the enzyme without affecting its expression. Compared to vehicle-treated cells, the HDAC6 mRNA levels were lower (by ∼40%) in cells exposed to SFN for 24 h (Fig. S4), in agreement with the complete inhibition of cell proliferation at this time point (Fig. 1D) and the previously reported decrease in HDAC protein levels following 24 h- or 48 h-treatemnt with SFN in prostate cancer cells (Clarke et al., 2011). Most importantly, the protein levels of HDAC6 did not change when the cells were exposed to SFN for 4 h (Fig. S5), which is the treatment time used in most of our experiments. The SFN-mediated increase in acetylation of α-tubulin and the inhibition of mTOR were slightly diminished upon overexpression of HDAC6, in agreement with the small increase in α-tubulin acetylation upon HDAC6 overexpression (Fig. 2C). The selective HDAC6 inhibitor tubastatin caused a profound increase in the acetylation of α-tubulin; however, in contrast to SFN, tubastatin only slightly affected the levels of pT389 p70S6K and pS235/236 S6 (Fig. 2D).

Inhibition of class I HDACs (HDAC1/2/3) suppresses the activity of mTOR in neonatal rat ventricular myocytes (Morales et al., 2016) and leukemia cell lines (Nishioka et al., 2008). Treatment with SFN increases the levels of acetylated histones H3 and H4 (Myzak et al., 2006), suggesting inhibition of class I HDACs. Therefore, we next tested the possibility that class I HDACs might be also involved in the SFN-mediated inhibition of mTOR. Treatment with the selective HDAC1/3 inhibitor MS-275 increased the acetylation of histones H3 and H4 (Fig. 2E), confirming the inhibitory effect of the compound. However, MS-275 had no effect on the levels of pT389 p70S6K and pS235/236 S6 either in the absence or in the presence of SFN (Fig. 2E). Next, we tested the effect of m-carboxycinnamic acid bis-hydroxamide (CBHA), another HDAC1/3 inhibitor, which also inhibits HDAC6 (Kumar et al., 2018). Similar to MS-275, CBHA caused a concentration-dependent hyperacetylation of histones H3 and H4 (Fig. 2E). In contrast to MS-275, which did not affect the levels of acetylated tubulin, CBHA increased tubulin acetylation. These results confirmed that MS-275 inhibits HDAC1/3 without affecting HDAC6, whereas CBHA inhibits HDAC1/3 as well as HDAC6. Most importantly, in contrast to MS-275, treatment with CBHA reduced the levels of pT389 p70S6K and pS235/236 S6 under both vehicle- as well as SFN-treatment conditions. Together, the results suggest that, under these experimental conditions, inhibition of HDAC6, but not HDAC1/3 is involved in the mTOR inhibition by SFN. However, HDAC6 inhibition alone is not sufficient to inhibit mTOR to the same extent as SFN. We conclude that SFN inhibits HDAC6 and decreases the catalytic activity of AKT, and this partially explains the mechanism by which SFN inhibits mTOR.

## Conflict of interest

We wish to confirm that there has been no financial support for this work that could have influenced its outcome. A.T.D.-K. is a member of the scientific advisory board for Evgen Pharma.

## Appendix Supplementary materials

Image, application 1
