# Supplementary material for: The isothiocyanate sulforaphane inhibits mTOR in an NRF2-independent manner
Source: Phytomedicine. 2021 Jun;86:None. doi: 10.1016/j.phymed.2019.153062 (PMC8106549; doi:10.1016/j.phymed.2019.153062)
Supplement: Supplementary file 1 [file mmc1.pdf]

Supplemental Material

The isothiocyanate sulforaphane inhibits mTOR in an NRF2-independent manner

Ying Zhang[a], Amy Gilmour[a], Young-Hoon Ahn[b], Laureano de la Vega[a], and Albena T. Dinkova-Kostova[a,c,*]

[a]Jacqui Wood Cancer Centre, Division of Cellular Medicine, School of Medicine, University of Dundee, Dundee, DD1 9SY Scotland, UK

[b]Department of Chemistry, Wayne State University, Detroit, MI, USA

[c]Department of Pharmacology and Molecular Sciences and Department of Medicine, Johns Hopkins University School of Medicine, Baltimore, MD 21205, USA

[*]Corresponding author: Albena T. Dinkova-Kostova, Jacqui Wood Cancer Centre, Division of Cellular Medicine, Ninewells Hospital and Medical School, James Arrott Drive, Dundee DD1 9SY, United Kingdom. Tel. (+44)-1382-383386; Email: a.dinkovakostova@dundee.ac.uk

Supplemental Materials and Methods

Materials

S-4 was synthesized according to the scheme below as previously described in detail (Zhang et al., 2014).

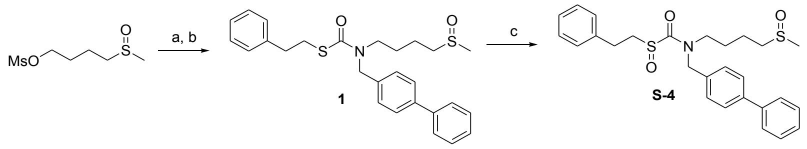

Reagents and conditions: (a) 4-Phenylbenzylamine, EtOH, 70°C; (b) *S*-phenylethyl chlorothioformate, DIPEA, CH_2Cl_2, 0°C; (c) *m*-CPBA, CH_2Cl_2, -78°C. ^{1}H-NMR ($CDCl_3$) δ 7.58 (*t*, J = 8.4, 4H), 7.45 (*q*, J = 6.6, 2H), 7.39-7.16(*m*, 8H), 4.92-4.56 (*m*, 2H), 3.76-3.02 (*m*, 6H), 2.70-2.63 (*m*, br, 2H), 2.55 (*d*, J = 1.6, 3H), 1.85-1.74 (br, 4H); ^{13}C-NMR ($CDCl_3$) δ 169.30, 168.86, 141.71, 141.50, 140.54, 140.38, 138.56, 138.49, 134.59, 134.09, 129.15, 129.12, 129.10, 129.09, 129.05, 128.97, 128.92, 128.07, 128.03, 128.01, 127.93, 127.84, 127.30, 127.19, 127.1553.89, 53.79, 53.31, 53.28, 53.14, 51.32, 51.30, 49.53, 49.50, 48.07, 48.03, 45.19, 45.13, 38.88, 38.84, 28.92, 28.23, 28.13, 26.27, 26.21, 20.11, 20.07, 19.98, 19.87. ESI-HRMS (M+Na) calculated for $C_{27}H_{31}NO_3NaS_2$ 504.1643, found *m/z* 504.1650.

Quantitative real-time PCR

The primers and probe (TaqMan® Gene Expression Assays) used to measure the mRNA levels for HDAC6 were from Life Technologies (Hs00195869_m1). U2OS cells seeded in 6-well plates were exposed to vehicle (0.1% DMSO) or SFN (20 µM) for the indicated periods of time. Cells were lysed and total RNA was extracted using RNeasy Kit (Qiagen Ltd.), and 500 ng total RNA was reverse transcribed into cDNA with Omniscript Reverse Transcription Kit (Qiagen Ltd.). Real-time PCR was performed on QuantStudio 5 Real-Time PCR System. The data were normalized using β-actin (human ACTB, Life Technologies, Hs01060665_g1) as an internal control.

Reference: Zhang, Y., Dayalan Naidu, S., Samarasinghe, K., Van Hecke, G.C., Pheely, A., Boronina, T.N., Cole, R.N., Benjamin, I.J., Cole, P.A., Ahn, Y.H., Dinkova-Kostova, A.T., 2014. Sulphoxythiocarbamates modify cysteine residues in HSP90 causing degradation of client proteins and inhibition of cancer cell proliferation. Br. J. Cancer 110, 71-82.

Supplemental Figures

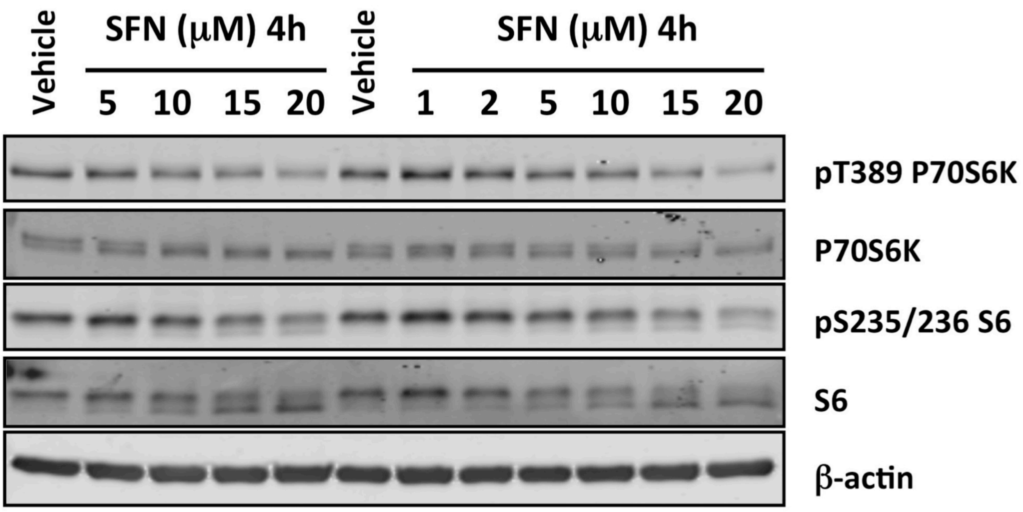

Figure S1. Sulforaphane (SFN) inhibits mTOR in a concentration-dependent manner. Immunoblotting analysis of phosphorylated p70S6K (T389), p70S6K, phosphorylated S6 (S235/236), and S6 in lysates from U2OS cells, which had been treated with vehicle (0.1% DMSO) or the indicated concentrations of SFN for 4 hours. The levels of β-actin served as a loading control.

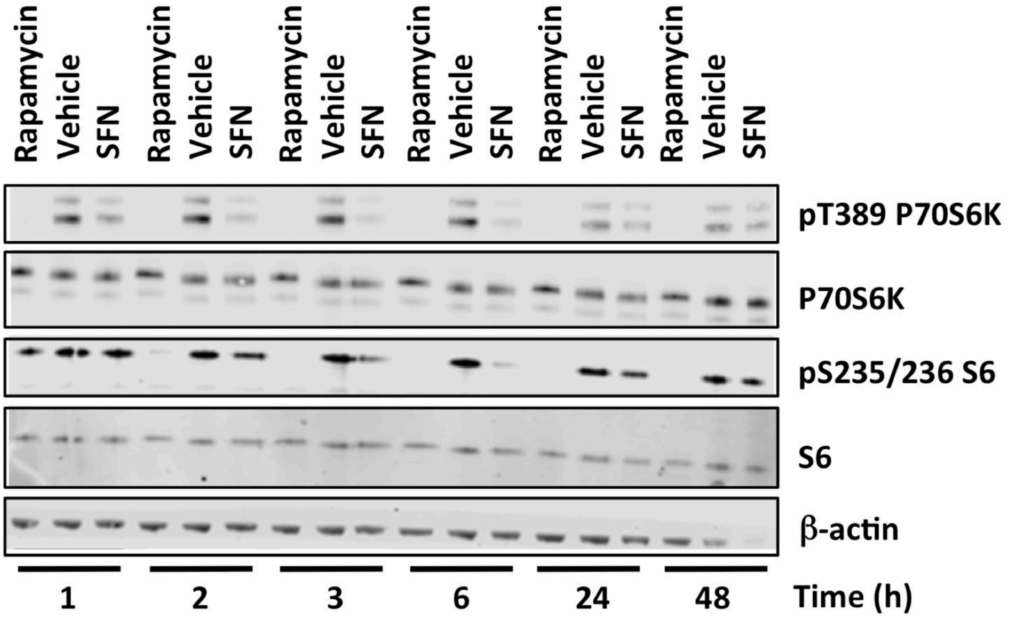

Figure S2. Persistent inhibition of mTOR by sulforaphane (SFN).
Immunoblotting analysis of phosphorylated p70S6K (T389), p70S6K, phosphorylated S6 (S235/236), and S6 in lysates from U2OS cells, which had been treated with vehicle (0.1% DMSO), rapamycin (20 nM), or SFN (20 μM) for the indicated periods of time. Proteins were resolved on a precast Bis-Tris 4–12% polyacrylamide gel (ThermoFisher Scientific) using MOPS running buffer. The levels of β-actin served as a loading control.

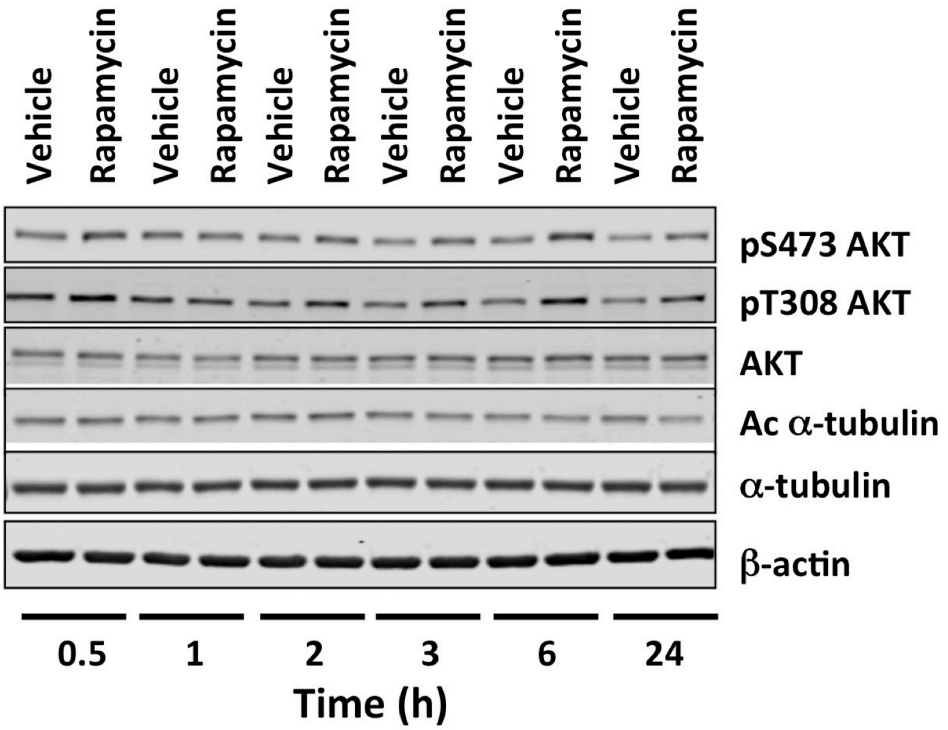

Figure S3. Similar to SFN, rapamycin causes an increase in the phosphorylation of AKT, but unlike SFN, rapamycin does not alter the acetylation of α-tubulin, suggesting that it does not inhibit HDAC6. U2OS cells were treated with vehicle (0.1% DMSO) or rapamycin (20 nM) for the indicated periods of time. Immunoblotting analysis of whole-cell lysates was used to determine the levels of: phosphorylated AKT (S473 and T308), AKT, acetylated α-tubulin (K40), and α-tubulin. The levels of β-actin served as a loading control.

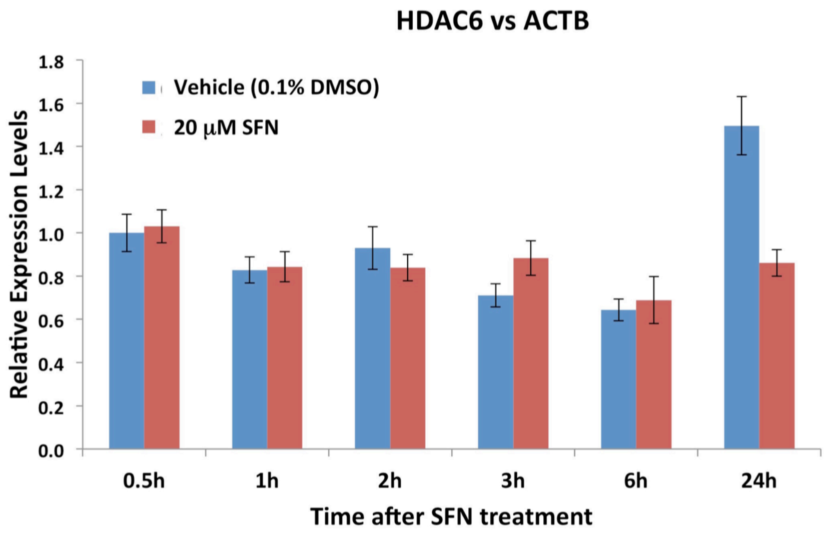

Figure S4. Short-term exposure to sulforaphane does not change the mRNA levels for HDAC6. U2OS cells were treated with vehicle (0.1% DMSO) or SFN (20 μM) for the indicated periods of time. Cells were lysed, total RNA was extracted and reverse transcribed into cDNA. The mRNA levels for HDAC6 were determined using quantitative real-time PCR. The data were normalized using β-actin (ACTB) as an internal control.

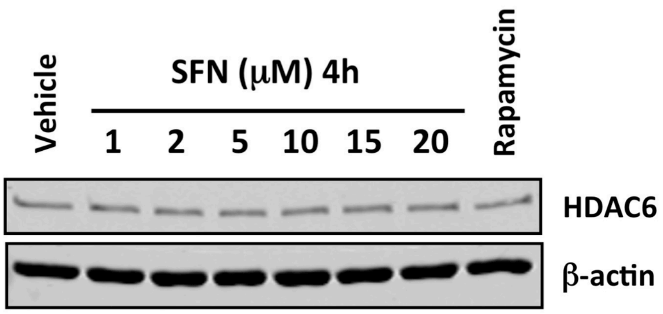

Figure S5. Short-term exposure to sulforaphane does not change the protein levels of HDAC6. Immunoblotting analysis of HDAC6 in lysates from U2OS cells, which had been treated with vehicle (0.1% DMSO), rapamycin (20 nM), or the indicated concentrations of SFN for 4 hours. The levels of β-actin served as a loading control.
